# Supplementary material for: Polymyxin Induces Significant Transcriptomic Perturbations of Cellular Signalling Networks in Human Lung Epithelial Cells
Source: Antibiotics (Basel). 2022 Feb 24;11(3):307. doi: 10.3390/antibiotics11030307 (PMC8944768; doi:10.3390/antibiotics11030307)
Supplement: Supplementary file 1 [file antibiotics-11-00307-s001.zip › antibiotics-1593513-supplementary/Figure S1-S2.pdf]

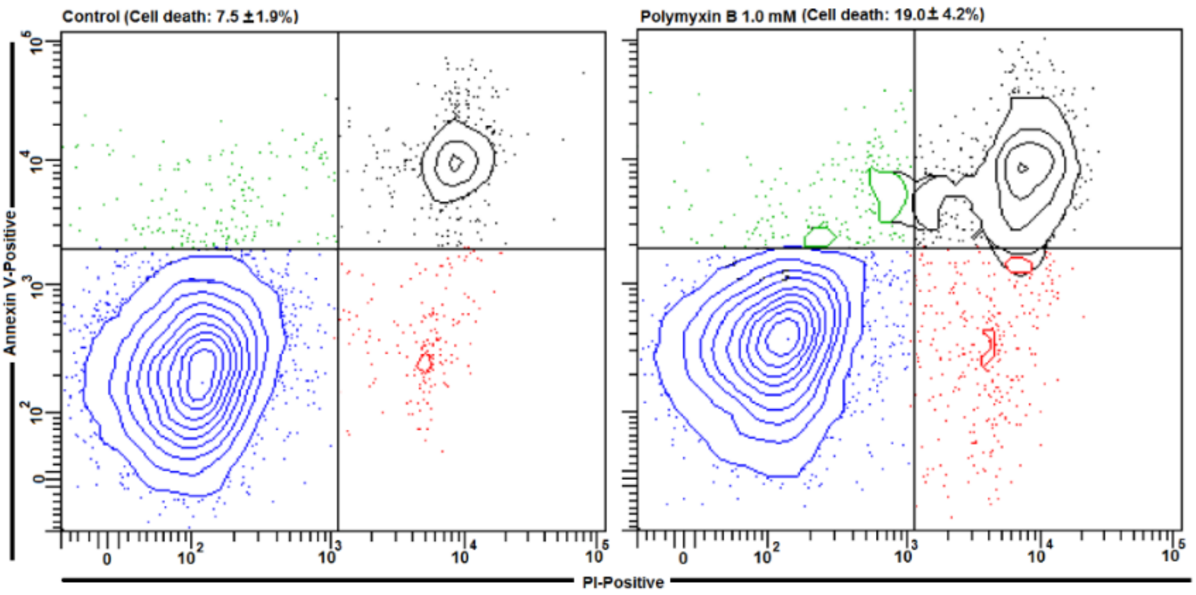

Figure S1. A549 cell viability as measured by flow cytometry. Representative dot plots of Annexin-V-Alexa fluor 488 and PI staining. A549 cells were treated with 1.0 mM polymyxin B (treatment group) or Milli-Q water (control group) for 24 h (n = 3, mean ± SD).

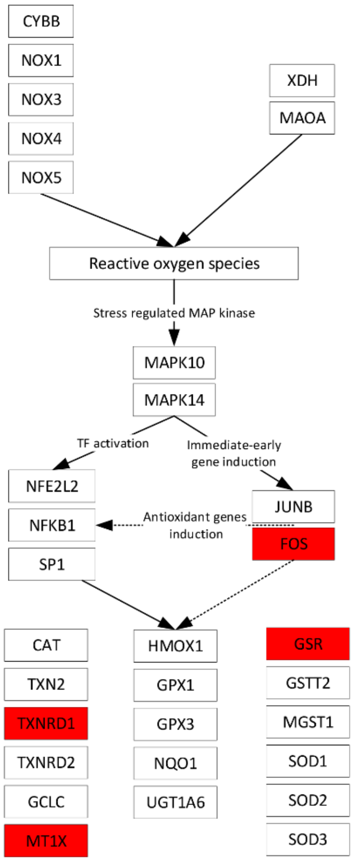

Figure S2. Activation of the antioxidant network system in A549 cells following the treatment with polymyxin B. Genes in red were up-regulated.
